# Adverse effects and discontinuation rates for darifenacin in overactive urinary bladder: a systematic review and meta-analysis of randomized controlled trials

**DOI:** 10.1007/s00210-025-04806-4

**Published:** 2026-01-09

**Authors:** Vineesha Veer, Felicity Smith, Anna Mae Scott, Christian Moro

**Affiliations:** 1 Centre for Urology Research, Faculty of Health Sciences and Medicine, Bond University, Gold Coast, QLD 4226 Australia; 2 Nuffield Department of Population Health, University of Oxford, Oxford, UK; 3 Warwick Medical School, University of Warwick, Coventry, UK

**Keywords:** Urinary bladder, Adherence, Bladder disease, Acetylcholine, Muscarinic receptor

## Abstract

The aim of this systematic review and meta-analysis was to identify the discontinuation rates, and incidence of adverse effects (AEs) for patients on prescribed oral darifenacin for the treatment of overactive bladder (OAB). PubMed, Embase, and Cochrane CENTRAL were searched for randomized controlled trials. A risk of bias assessment and a Grading of Recommendations Assessment, Development and Evaluation were used to assess the certainty of evidence. The primary outcome was OAB patient discontinuation and AEs in darifenacin and placebo groups, and reporting was performed using the Preferred Reporting Items for Systematic Reviews and Meta-Analyses (PRISMA). Seven studies of 2381 participants were included. The most reported AEs were dry mouth and constipation. There were more participants with dry mouth in the darifenacin group compared to the placebo (*p* < 0.0001, moderate level of certainty) and a dose–response pattern was observed. There was a higher rate of constipation in the darifenacin group than in the placebo group (*p* < 0.0001, high level of certainty), with evidence of a dose–response pattern. There were no differences between the darifenacin group and the placebo group in total discontinuations (RR 0.93, 95% CI 0.72–1.20) or undefined discontinuations (RR 0.85, 95% CI 0.54–1.33). Studies were generally rated at low or unclear risk of bias for most domains. While patients prescribed darifenacin do experience a higher rate of side-effects, which increases with dose, they seem to be tolerated as both the intervention and placebo groups reported similar rates of discontinuation.

**Supplementary Information:**

The online version contains supplementary material available at 10.1007/s00210-025-04806-4.

## Introduction

Lower urinary tract symptoms (LUTS) are a broad category of symptoms that occur during storage and voiding. Overactive bladder (OAB) is a subgroup of storage LUTS, affecting 11–20% of the population, placing a substantial burden on healthcare services (Chuang et al. 2019; Irwin et al. 2006). The most widespread definition of OAB is provided by the International Continence Society, which characterizes OAB by urinary urgency, typically accompanied by an increase of urinary frequency in the daytime and nocturia, when there is no other obvious pathology or confirmed infection (Haylen et al. 2010). In some cases, this condition could also include the presence of urgency urinary incontinence. As such, this condition can affect a patient’s quality of life. Due to the constant trips to the bathroom, activities and routines such as social events, employment, physical activity, and sleep is disrupted. Though the pathophysiology of OAB is not well understood, OAB symptoms are thought to occur due to spontaneous contractions of the detrusor muscle during the filling phase. This is likely a result of abnormal increases in detrusor M3 stimulation from acetylcholine, released from the parasympathetic nerves or the nearby urothelium and lamina propria (Moro et al. 2011).

Common first-line antimuscarinic treatments approved for OAB treatment include darifenacin, fesoterodine, oxybutynin, propiverine, solifenacin, tolterodine, and trospium (Mostafaei et al. 2020). However, studies have reported low adherence rates, with 70% of newly diagnosed OAB patients discontinuing their antimuscarinic treatment within the first 12 months of initiation (Vouri et al. 2019). Furthermore, the most recent systematic review conducted by Yeowell et al. (2018) on antimuscarinic persistence for OAB patients have echoed these low adherence rates suggesting a range of 12–25% of patients adhering to their treatment after one year. Though it is not fully understood why adherence is so low, it is suggested to be due to a lack of expected benefits or an increase in bothersome adverse events, including dry mouth, constipation, or headaches (Kim et al. 2017).

While there are multiple options within this class, darifenacin remains one of the newer-generation prescribed medications for OAB (Yamada et al. 2012; Aparasu et al. 2020), presenting an importance to assessing the evidence on its adherence rates and adverse effects (Veer et al. 2025). While other reviews have investigated the adherence to antimuscarinics in general (Yeowell et al. 2018; Veenboer and Bosch 2014), this is the first systematic review and meta-analysis of randomized controlled trials specifically focused on darifenacin. The aim of this systematic review and meta-analysis was to identify the adherence rates, incidence, and type of adverse events for patients on prescribed oral darifenacin for the treatment of overactive bladder.

## Methods

The reporting of systemic review was completed in compliance with Preferred Reporting Items for Systematic Reviews and Meta-Analyses (PRISMA) statement. An a priori protocol was submitted to PROSPERO (CRD42024519450) on April 2, 2024, with the initial searches performed on April 4, 2024.

### Inclusion and exclusion criteria

We included studies with patients of any age, gender, or condition, presenting with OAB as defined by study authors. Studies with participants exhibiting OAB symptoms that arose from a recent surgery were excluded. Also, studies with patients being treated for coexisting bladder conditions alongside OAB, or taking darifenacin as a combination therapy with other medications to treat OAB were excluded. We included studies of any dose of oral formulations of darifenacin, both extended release and intermediate release. We included studies with a placebo comparator. Randomized controlled trials were selected because they are considered one of the most reliable types of studies of the effectiveness of interventions in the evidence hierarchy. To be included, studies had to report patient discontinuation of both the intervention group and the comparator group (primary outcome). Secondary outcomes included patient withdrawal rates from the study and adverse events. Randomized controlled trials of any design were included. Studies reported only as an abstract (e.g., a conference abstract) were excluded.

### Search strategy and information sources

The search string, comprised of keywords and MeSH terms, was designed in PubMed and translated to other databases using Polygot Search Translator (sr-accelerator.com/polyglot). The search string in full is provided as Supplementary Material 1. Search concepts included Darifenacin AND Overactive Bladder AND Randomised Control Trial. PubMed, Embase, and Cochrane CENTRAL were searched for studies from inception until April 4, 2024. Complete published articles in English were included with no date of publication imposed. Trials available as an abstract were also included, if all information for extraction was reported. A forward and backward citation search was conducted on the 31 May 2024 using SpiderCite (sr-accelerator.com/spidercite), with additional articles screened for inclusion.

### Selection process and screening

All title and abstract screening, full-text screening, and data extraction were conducted independently by two authors (V.V. and F.S.), with discrepancies resolved via consensus or by a third author (C.M.). Following screening of articles for inclusion, we also consulted experts for other public reports. All screening was conducted using Covidence (Veritas Health Innovation, Melbourne, Australia).

### Data extraction

A Table of Characteristics, Outcomes form and Risk of Bias data extraction forms were created using Microsoft Excel 2024 (Microsoft, Redmond, WA, USA). These forms have been pre-piloted and validated for its use for extraction in other studies (Moro et al. 2024, 2022). Data extraction was conducted independently by two authors (V.V. and F.S.), with discrepancies resolved by consensus or by referring to the third author (C.M.). We contacted study corresponding investigators for six of the seven included studies to obtain missing data. We thank Chapple et al. (2007) for the email response and clarification regarding data included in their included manuscript.

### Risk of bias

The assessment of the risk of bias was conducted independently by two authors (V.V. and F.S.). Discrepancies were resolved by consensus or reference to another author (C.M.). We used the Cochrane Collaboration’s Risk of Bias Tool 1 (Higgins and Green. 2019). We prespecified in the protocol that we would use the Risk of Bias Tool 2; however, we chose to use the Cochrane Risk of Bias Tool 1, because it enabled us to evaluate the potential biases arising from conflict of interest or funding sources (under domain 7, other bias). The following domains were assessed: (1) random sequence generation, (2) allocation concealment, (3) blinding of participants and personnel, (4) blinding of outcome assessment, (5) incomplete outcome data, (6) selective outcome reporting, and (7) other bias (focusing on potential for biases due to funding or conflict of interest). Each potential source of bias was graded as low, unclear, or high. A quote from the relevant trial supported the judgement made for each risk of bias rating.

### GRADE assessment

The assessment of the quality of evidence using Grading of Recommendations, Assessment, Development, and Evaluations (GRADE) using GRADEpro (McMaster University and Evidence Prime, Ontario, Canada) was also conducted (V.V.), and the profile is attached as Supplementary Material 2.

### Data synthesis

Review Manager Software version 5.4 (Cochrane Collaboration, London, UK) was used for all effect size analyses. As all outcomes were dichotomous, we calculated risk ratios with 95% confidence intervals (CI). The *I*^2^ statistic was used to measure heterogeneity. A random effects model was used for all meta-analyses conducted, as heterogeneity was anticipated. There was sufficient data for the meta-analysis of four outcomes: total withdrawals, undefined withdrawals, and incidences of two common adverse events: dry mouth and constipation. Total adverse effects and other specific adverse effects were not meta-analyzable and were reported narratively. The individual was the unit of analysis.

### Subgroup and sensitivity analysis

We prespecified the following subgroup analyses: by dose, type of comparator, and timing of the outcome. Therewere sufficient data to conduct subgroup analysis by dose and timing of the outcome. As reported in the “Inclusion and exclusion criteria” section, we narrowed the included intervention only to darifenacin, and thus did not conduct a subgroup analysis by type of antimuscarinic. For all studies, the comparator was placebo, precluding analysis by comparator type. We had intended to perform sensitivity analyses by comparing the inclusion and exclusion of studies with an overall rating of high risk of bias. Due to the change from Risk of Bias Tool 2 to Risk of Bias Tool 1, this was reconceptualized as being rated in three or more domains at high risk of bias. However, since none of the included studies was rated as having a high risk of bias in three or more domains, we did not carry out the sensitivity analyses.

## Results

### Literature search results

**Fig. 1:**
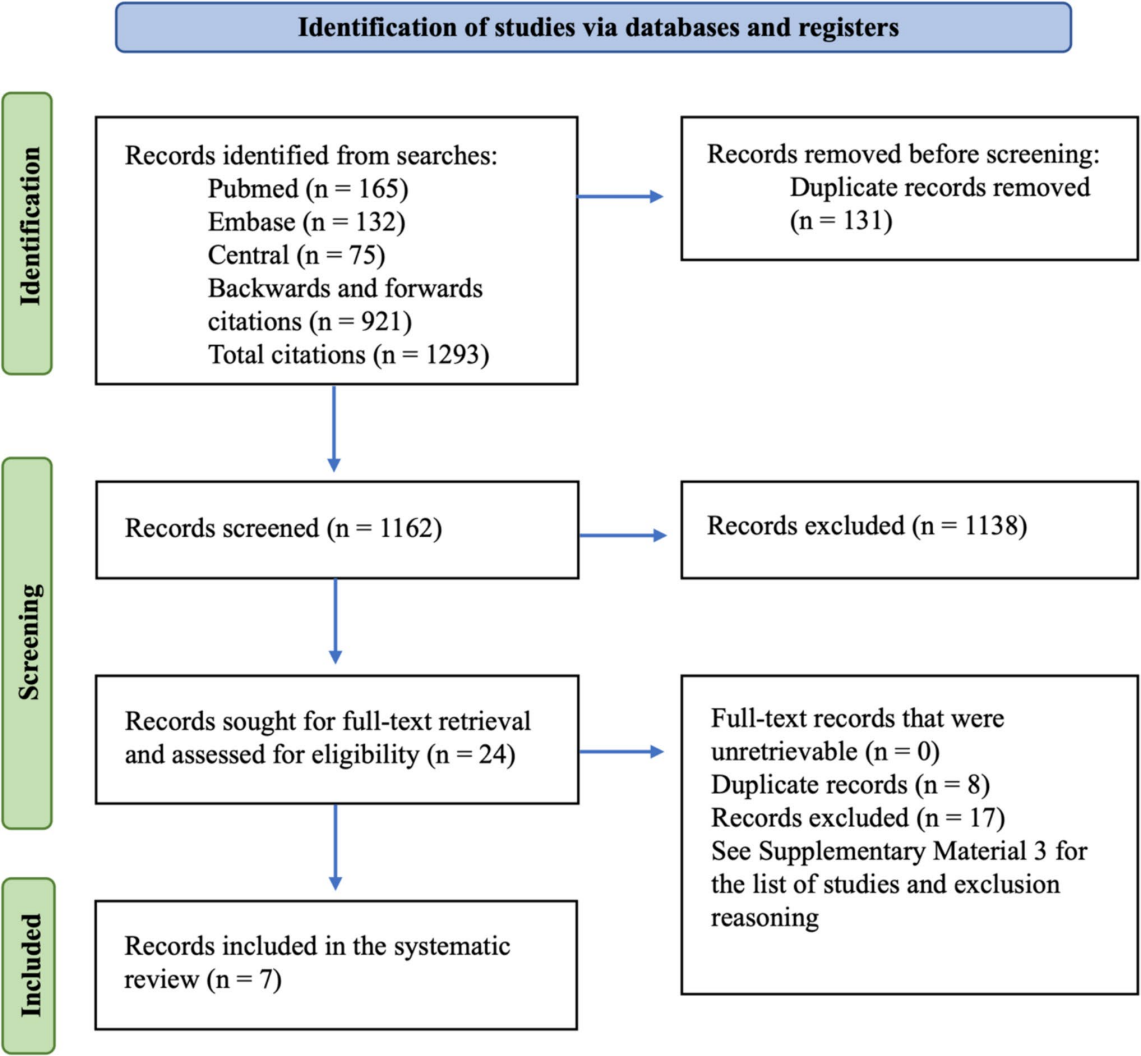
PRISMA flow diagram. Supplementary Material 3 contains the references and reasons for full-text excluded articles. PRISMA, Preferred Reporting Items for Systematic Reviews and Meta-Analyses; RCT, randomized controlled trial

Searches across several databases resulted in a total of 372 articles, with 921 additional articles added through a manual backward and forward citation search (Fig. 1). From this search, 131 duplicate records were then removed. Of the 1162 title and abstract records screened, 1138 were excluded and 24 full-texts were obtained for full-text screening. Seventeen texts were excluded during the full-text screening stage and a list of excluded studies with the reasoning for exclusion is available as Supplementary Material 3. Seven studies were included in the results; all were included from the database searches, as none was included from the forward and backward citation search.

### Included studies and characteristics

Seven studies of 2381 participants in aggregate were included into the analysis (Table 1). Two studies were conducted in the USA (Zinner et al. 2005, 2006), one in the UK (Cardozo and Dixon 2005), one across eight countries (Hill et al. 2006), one across seven countries (Chapple et al. 2007), one was conducted across three countries (Haab et al. 2004), and one study conducted in two countries (Steers et al. 2005). The most common duration for follow-up was 12 weeks (four studies), with two studies having a follow-up duration of 2 weeks and one study included two timepoints at 2 and 12 weeks. Study populations ranged from 72 to 561 participants. Of the 2381 participants, 1995 are female and 386 males. The author’s criterion for OAB was similar for five studies, with symptoms of OAB being present for at least 6 months (urgency or frequency) before the initiation of the study. The remaining two studies’ criteria included OAB symptoms; however, one did not specify a time period, and another included OAB patients’ need to have undergone cystometry in the past year.

**Table 1:** Description and characteristics of studies included in the review. RCT, randomised controlled trial; ER, extended release; IR, immediate release

Author Year Location	Study design Follow-up	No. of participants (*N* each arm)	Gender	Age (years)	Participant eligibility for trial	Darifenacin intervention Dose, frequency, duration CR, controlled release IR, immediate release	Comparator(s) Dose, frequency, duration
Cardozo and Dixon (2005) UK	Parallel-group RCT study 2 weeks	72 total (36 control, 36 darifenacin)	50% females, 50% males	Mean 54 Range	Symptoms of urgency for ≥ 6 months. Within the 14-day run in period, had 4 episodes of urgency within 24 h for 5 days	A1: Darifenacin (oral), 30 mg ER, 1× daily, 2 weeks	A2: Placebo (details NR), 1× daily, 2 weeks
Chapple et al (2007) USA, Poland, South Africa, Hungary, Sweden, UK, Germany	Parallel-group RCT study 12 weeks	399 total (133 control, 266 darifenacin)	76.7% females, 22.3% males	Mean 72 Range 64–89	Symptoms of OAB	A1: Darifenacin (oral), 7.5 mg ER, 1× daily, 12 weeks A2: Darifenacin (oral), 7.5 mg ER, 1× daily, 2 weeks, then up titration to darifenacin (oral), 15 mg CR, 1× daily, 10 weeks	A3: Placebo (NR)
Haab et al (2004) Australia, UK, France	Parallel-group RCT study 12 weeks	561 total (164 control, 53 3.75 mg darifenacin, 229 7.5 mg darifenacin, 115 15 mg darifenacin)	85% females, 15% males	Range 19–88 years	Symptoms of OAB for ≥ 6 months. Includes incontinence urgency (5–50 episodes per week), frequency (8 voids per 24 h) and urgency	A1: Darifenacin (oral), 3.75 mg ER, 1 × daily, 12 weeks A2: Darifenacin (oral), 7.5 mg ER, 1× daily, 12 weeks A3: Darifenacin (oral), 15 mg ER, 1× daily, 12 weeks	A4: Placebo tablets, 1× daily, 12 weeks
Hill et al. (2006) Belgium, Denmark, Israel, Norway, Poland, Sweden, The Netherlands, UK	RCT study 12 weeks	439 total (109 control, 108 7.5 mg darifenacin, 107 15 mg darifenacin, 115 30 mg darifenacin)	85.4% females, 14.6% males	Range 21–88 years	Symptoms of urgency for ≥ 6 months. Includes urge incontinence (≥ 4 episodes), frequency (mean of 8 voids per 24 h) and urgency (strong urge to desire)	A1: Darifenacin (oral), 7.5 mg ER, 1× daily, 12 weeks A2: Darifenacin (oral), 15 mg ER, 1× daily, 12 weeks A3: Darifenacin (oral), 30 mg ER, 1× daily, 12 weeks	A4: Placebo (details NR), 1× daily, 12 weeks
Steers et al (2005) Canada USA	RCT study 2 weeks and 12 weeks	395 total, 127 control, 268 darifenacin	84% females, 16% males	Control mean 58.5, darifenacin mean 57.5	Aged ≥ 18 years with symptoms of urgency for ≥ 6 months	A1: Darifenacin (oral), 7.5 mg ER, 1× daily, 12 weeks A2: Darifenacin (oral), 7.5 mg ER, 1× daily, 2 weeks, then uptitration to darifenacin (oral), 15 mg ER, 1× daily, 10 weeks	A3: Placebo (details NR), 1× daily, 12 weeks A4: Placebo (details NR), 1× daily, 2 weeks then pseudo uptitration to placebo (details NR), 1× daily, 10 weeks
Zinner et al (2006) USA	RCT study 12 weeks	439 total, 225 control, 214 darifenacin	86.1% females and 13.9% males	Mean 59.1	Aged ≥ 18 years with a history of OAB for ≥ 6 months and average ≥ 1 urge incontinence episodes/day; ≥ 8 micturitions/day; ≥ 4 urgency episodes/day and mean warning time of ≥ 15 min during 12 consecutive hours	A1: Darifenacin (oral), 15 mg ER, 1× daily, 12 weeks	A2: Placebo (details NR), 1× daily, 12 weeks
Zinner et al (2005) USA	Crossover RCT study 2 weeks	76 total, 76 control, 76 darifenacin	93.4% females and 6.6% males	Mean 59.9 Range 33–84	OAB patients who had undergone cystometry in previous 12 months. Urge incontinence (≥ 4 significant incontinent episodes/week) and urinary frequency (≥ 8 voids/day)	A1: Darifenacin (oral), 15 mg ER, 1× daily, 2 weeks A2: Darifenacin (oral), 30 mg ER, 1× daily, 2 weeks A3: Darifenacin (oral), 15 mg IR, 3× daily, 2 weeks	A4: Placebo tablets, 1× daily, 2 weeks

### Risk of bias assessment

**Fig. 2:**
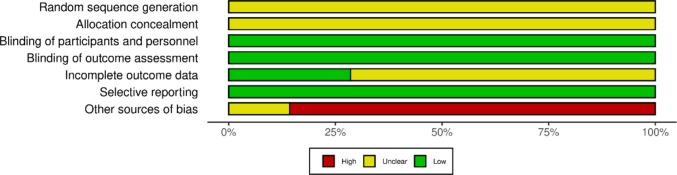
Risk of bias graph. Presented as percentages, this provides the review authors’ judgements about each risk of bias item across all included studies

Studies were at unclear risk of bias for randomization and allocation concealment due to unclear reporting. However, blinding of participants and personnel, of outcome assessors, and selective reporting were at low risk of bias. Incomplete outcome data was rated at unclear risk of bias for five of the seven studies, due to unclear reporting. Finally, six of the seven studies were rated at high risk of bias due to concerns about conflict of interest and funding sources. (Fig. 2, Supplementary Material 4).

### Adverse events

**Fig. 3:**
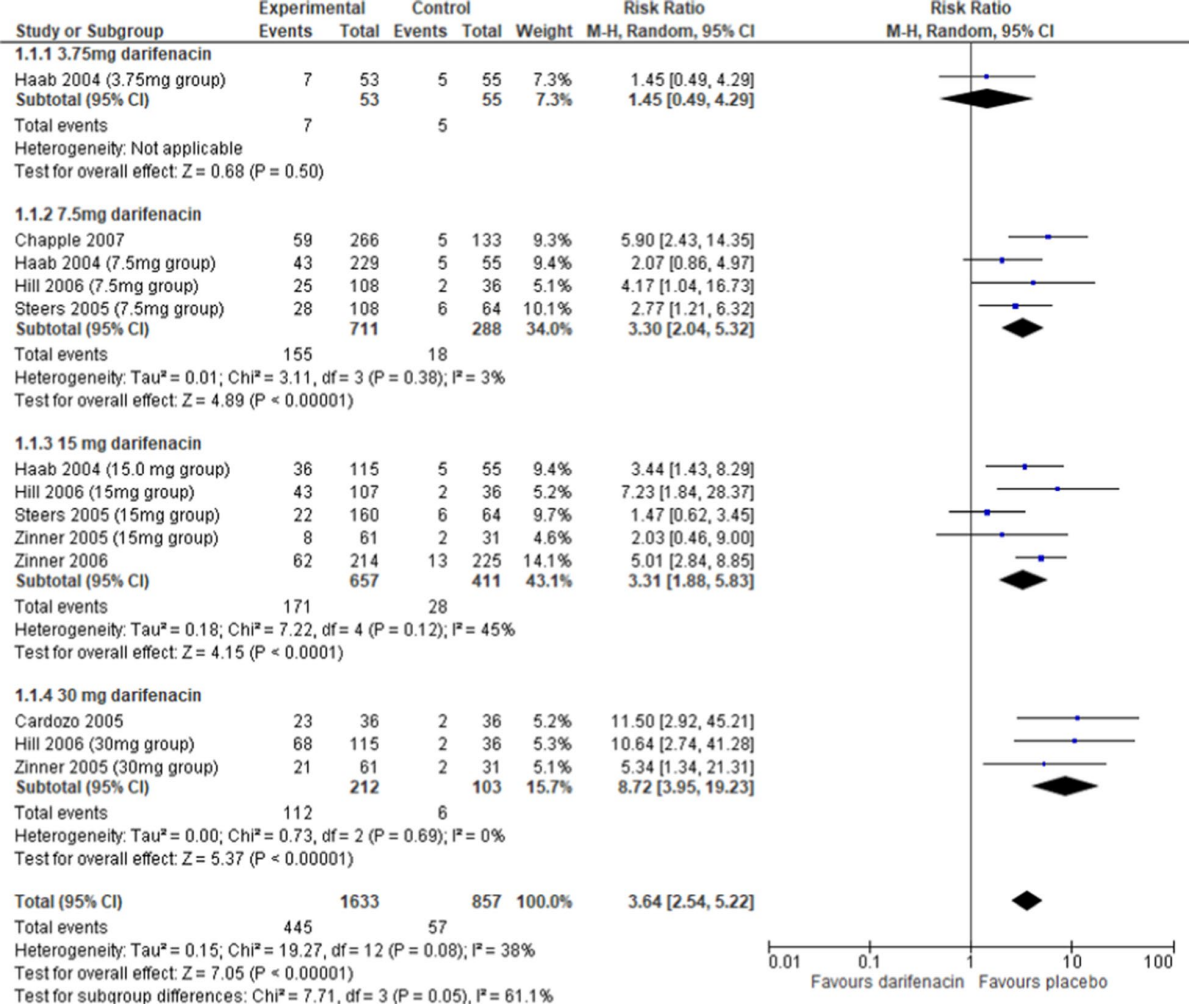
Dry mouth adverse event incidence of placebo and darifenacin, sub-grouped by dose. CI, confidence interval

Dry mouth and constipation were the most common adverse events in all seven studies (see Supplementary Material 10) and were able to be meta-analyzed. The overall adverse events were unable to be meta-analyzed, due to the lack of reporting of all adverse events by three of the seven included studies, and inconsistent reporting among the remaining four studies. Overall, there were more participants with dry mouth in the darifenacin than the placebo group (risk ratio 3.64, 95% CI 2.54–5.22, *I*^2^ = 38%, *p* < 0.0001, Fig. 3), with evidence of a dose–response pattern. The quality of the evidence for this outcome was moderate (Supplementary Material 2). There were also more participants reporting dry mouth at 7.5 mg, 15 mg, and 30 mg doses, but not at the 3.75 mg dose, where the difference between darifenacin and placebo was not significant, although the evidence at that dose consisted of only one study (risk ratio 1.45, 95% CI 0.49–4.29, Fig. 3). When sub-grouped by timepoint, there were more people with dry mouth in the darifenacin group than in the placebo group both at 2 weeks (risk ratio 5.17, 95% CI 1.94–13.77, *I*^2^ = 31%, *p* = 0.001) and at 12 weeks (risk ratio 3.42, 95% CI 2.32–5.06, *I*^2^ = 42%, *p* < 0.00001, Supplementary Material 5).

**Fig. 4:**
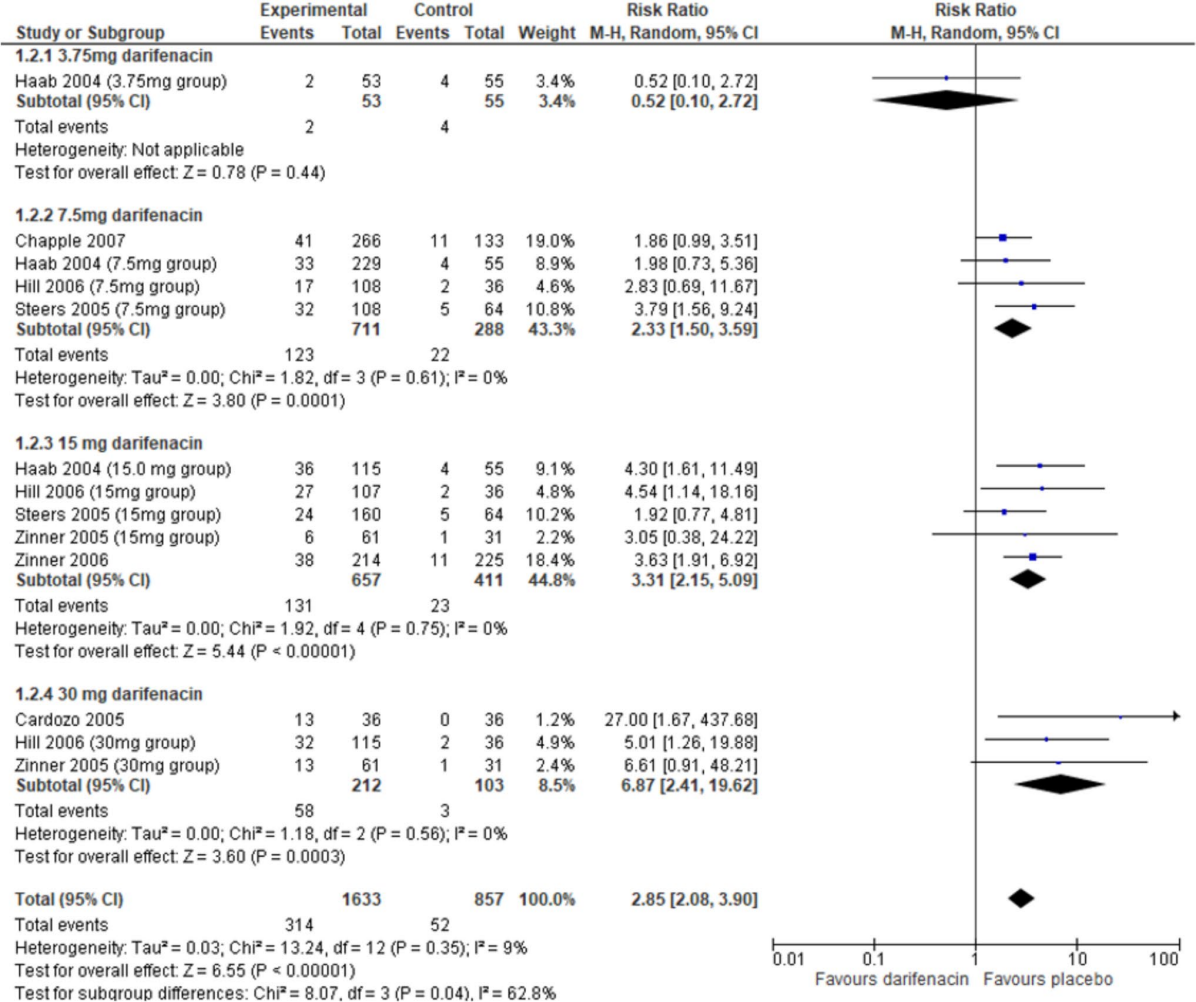
Constipation adverse event incidence of placebo and darifenacin, sub-grouped by dose. CI, confidence interval

Overall, more participants in the darifenacin group also experienced constipation than in the placebo group (risk ratio 2.85, 95% CI 2.08–3.90, *I*^2^ = 9%, *p* < 0.0001, Fig. 4), and the subgroup analysis by dose suggests evidence of a dose–response pattern. The quality of the evidence for this outcome was high (Supplementary Material 2). There was no difference between darifenacin and placebo at the 3.75 mg dose of darifenacin; however, this is a finding from a single study. The differences between darifenacin and placebo for the events of constipation were significant at the 7.5 mg dose, 15 mg dose, and 30 mg dose of darifenacin. When sub-grouped by timepoint at which constipation was reported, there were more people with constipation in the darifenacin group than in the placebo group both at 2 weeks (risk ratio 6.62, 95% CI 1.85–23.7, *I*^2^ = 0%, *p* = 0.004) and at 12 weeks (risk ratio 2.70, 95% CI 1.98–3.70, *I*^2^ = 7%, *p* < 0.00001, Supplementary Material 6).

### Discontinuation rates and undefined withdrawals from studies

**Fig. 5:**
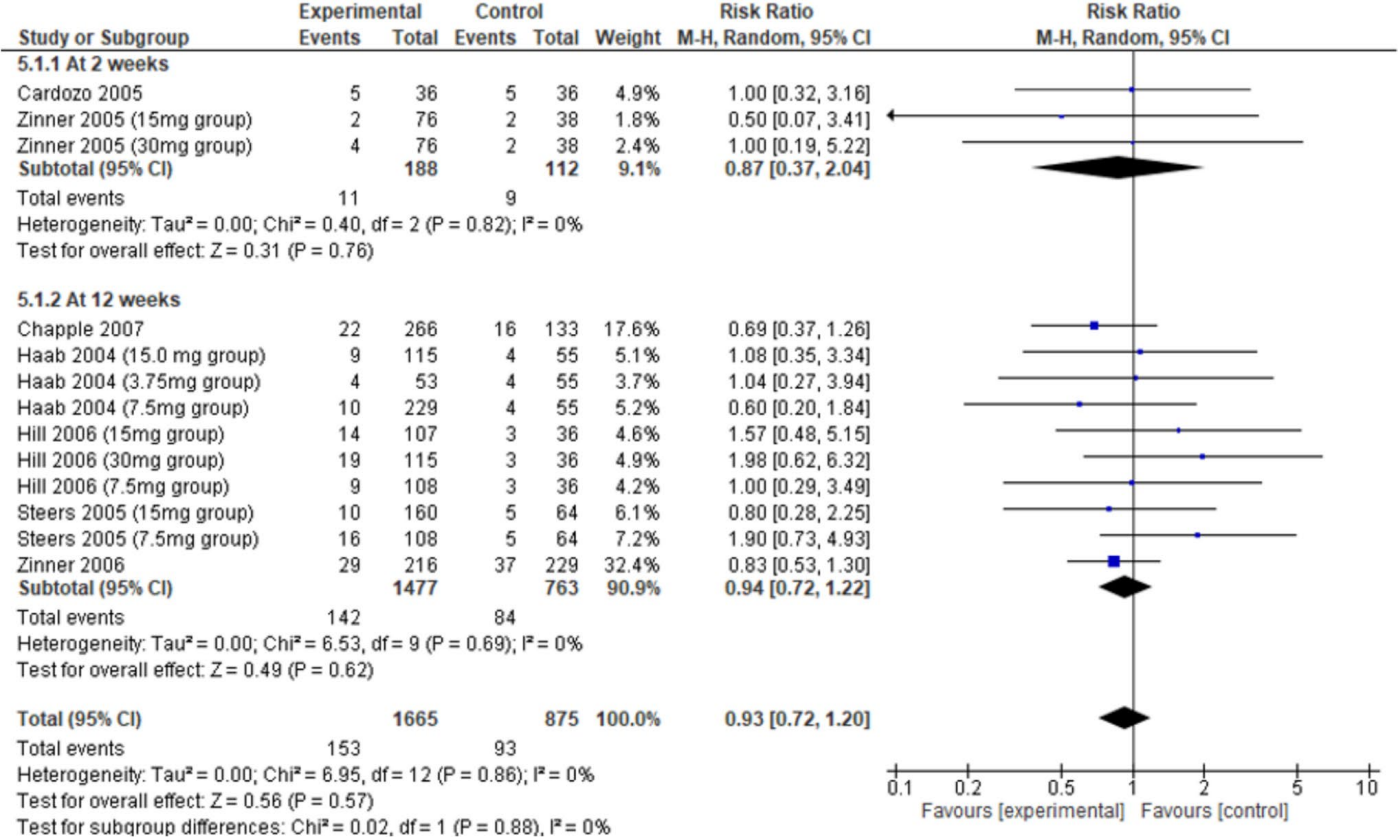
Total patient discontinuations of darifenacin and placebo, sub-grouped by timepoint. CI, confidence interval

The number of participants included across the seven studies in the assessment of discontinuation rates was 1663 participants in the darifenacin arms and 870 in the placebo arms (see Supplementary Material 11). Total discontinuation rates were reported across all seven studies and were able to be meta-analyzed. Overall, there were no differences between total patient discontinuations between the darifenacin or placebo groups overall (risk ratio 0.93, 95% CI 0.72–1.20, *I*^2^ = 0%,* p* = 0.57) or at any dose of darifenacin (Supplementary Material 8). When subgroup analysis was conducted by timepoint at which the total patient discontinuation was reported, there were no significant differences between the darifenacin group and the placebo group at either 2 weeks (risk ratio 0.87, 95% CI 0.37–2.04, *I*^2^ = 0%, *p* = 0.76) or at 12 weeks (risk ratio 0.94, 95% CI 0.72–1.22, *I*^2^ = 0%, *p* = 0.62, Fig. 5). The quality of this evidence is low (Supplementary Material 2). Out of the seven studies, six studies included discontinuations that were undefined by the authors. These were able to be meta-analyzed; there were no significant differences in undefined discontinuations between the darifenacin group and the placebo group overall (risk ratio 0.85, 95% CI 0.54–1.33, *I*^2^ = 0%, *p* = 0.47), or at any dose of darifenacin (Supplementary Material 8), with the quality of the evidence being low (Supplementary Material 2). The analysis of undefined patient discontinuations when sub-grouped by timepoint revealed no significant differences between the darifenacin group and the placebo group at either 2 weeks (risk ratio 0.74, 95% CI 0.21–2.61, *I*^2^ = 0%, *p* = 0.64) or at 12 weeks (risk ratio 0.86, 95% CI 0.53–1.40, *I*^2^ = 0%, *p* = 0.55).

## Discussion

This study provides insights into the prominent discontinuation rates and adverse effects for patients using darifenacin at 2-week and 12-week time points. Across the seven studies for both timepoints, adverse effects were higher in the darifenacin arms compared to the placebo, with dose–response patterns observed, while the discontinuation rates were similar. This suggests that the adverse events associated with this medication not only increase with dose but could also be tolerated, potentially due to the ability of the medication to relieve symptoms of OAB.

It was not possible to clearly identify patients’ reasons for discontinuation due to poor reporting. Six out of the seven studies did not provide substantial information on why a number of patients withdrew their participation. Furthermore, three out of seven studies did not provide enough information to assess the full degree of all adverse effects. For example, two studies did not explain why fewer participants were included in the safety and tolerability profiles compared to other results, and two studies did not provide the total adverse events for each arm. There are not only a lack of well-reported studies regarding the discontinuation and adverse effects of darifenacin, but a lack of studies investigating darifenacin-reported adherence and discontinuation, as shown by the inclusion of only seven studies in this review overall. Thus, a consistent issue identified across these studies assessing darifenacin as a treatment for OAB is the need to transparently report reasonings for decisions undertaken in clinical methodology, as well as the results obtained.

Though excluded based on our criteria, additional insights were also gained from reviewing studies that assessed the influence of darifenacin on healthy subjects. The study conducted by Kay et al. (2006) noted higher discontinuation and adverse events in the darifenacin group compared to the placebo group. There was no discontinuation across all groups in the Serra et al. (2005) study; however, higher rates of adverse events were noted in the darifenacin arms. As the discontinuation is higher in healthy subjects compared to studies with OAB participants, it can be inferred that this medication can provide relief specifically for OAB patient symptoms. However, it should also be noted that darifenacin continues to cause bothersome adverse effects in both OAB and healthy participants, decreasing its tolerability in a clinical setting. It is not clear why these adverse events occur, but previous *in vitro* laboratory studies have identified darifenacin as a potential insurmountable antagonist, suggesting a lack of specificity for the targeted M_3_ bladder receptors (Veer et al. 2023).

One important issue identified was the increased risk of bias due to issues from funding and conflict of interest. Six of the included studies were directly funded by interested pharmaceutical companies, while the remaining study (Cardozo and Dixon 2005) did not disclose a statement of funding. It is this potential for conflict of interest in included data, along with the limited provision of information for accurate discontinuation rates and adverse events, that creates difficulty in drawing conclusions.

### Deviation from the protocol

Initially, the submitted protocol (PROSPERO, CRD42024519450, April 2, 2024) outlined the aim to assess multiple antimuscarinics. The extremely large number of papers returned from the initial title and abstract search was unanticipated and as such, the study could not have been possible in the time allocated. To reduce the volume of papers that required full-text screening to a reasonable amount, only darifenacin was investigated. This change now meant that only 1162 papers required screening. An additional deviation was to utilize the Cochrane Risk of Bias Tool 1, instead of the Cochrane Risk of Bias Tool 2 as noted in the “Methods” section.

### Strengths and limitations

This review assessed randomized controlled trials because they are widely regarded as one of the most trustworthy study designs for the evaluation of the effectiveness of interventions in the evidence hierarchy. It became apparent during this study that to fully understand discontinuation, a broader approach could be applied, which incorporates qualitative studies, feedback from patients and written testimonies. Also, patient perceptions about the benefits of the medication may be informative. This systematic review also focused on studies in English. Future studies could benefit by incorporating publications from other languages, while also expanding the scope to include other commonly prescribed antimuscarinics for OAB, rather than solely darifenacin. This study systematically reviewed and meta-analyze discontinuation rates alongside the adverse effects of darifenacin in OAB patients, updating the current systematic literature reviews of antimuscarinic medication discontinuation rates. As darifenacin remains a widely used medication for OAB, this study addresses the ongoing need to assess compliance and adverse events to darifenacin within clinical settings.

## Conclusion

Participants prescribed darifenacin exhibited a higher incidence of adverse events of dry mouth (with moderate quality evidence) and constipation (with high-quality evidence). However, there were similar discontinuation rates in both the placebo and darifenacin arms (with low-quality evidence). The presence of undefined discontinuations within all studies presents challenges when identifying the precise reasons for many withdrawals and highlights the need for improved patient record-keeping for discontinuation within clinical trials.

## Supplementary Information

Below is the link to the electronic supplementary material.

ESM1 (DOCX 670 KB)

## Data Availability

All source data for this work (or generated in this study) are available upon reasonable request.
