# Supplementary material for: Adverse effects and discontinuation rates for darifenacin in overactive urinary bladder: a systematic review and meta-analysis of randomized controlled trials
Source: Naunyn Schmiedebergs Arch Pharmacol. 2026 Jan 9;399(6):8645–56. doi: 10.1007/s00210-025-04806-4 (PMC13086819; doi:10.1007/s00210-025-04806-4)
Supplement: Supplementary file 1 — (DOCX 670 KB) [file 210_2025_4806_MOESM1_ESM.docx]

**Supplementary Material 1**: Search strategy

**PubMed**

( "Darifenacin"[Title/Abstract])

AND

("Overactive Bladder"[Title/Abstract] OR "Overactive urinary bladder"[Title/Abstract] OR "Overactive detrusor"[Title/Abstract] OR "Urge incontinence"[Title/Abstract] OR "Neurogenic bladder"[Title/Abstract] OR "Detrusor hyperreflexia"[Title/Abstract] OR "Detrusor overactivity"[Title/Abstract] OR "urinary bladder, overactive"[MeSH Terms])

AND

("randomized controlled trial"[Publication Type] OR "controlled clinical trial"[Publication Type] OR "randomized"[Title/Abstract] OR "randomised"[Title/Abstract] OR "placebo"[Title/Abstract] OR "drug therapy"[MeSH Subheading] OR ("randomly"[Title/Abstract] OR "trial"[Title/Abstract] OR "groups"[Title/Abstract])) NOT ("animals"[MeSH Terms] NOT "humans"[MeSH Terms])

**Embase**

(Darifenacin:ti,ab)

AND

('Overactive Bladder':ti,ab OR 'Overactive urinary bladder':ti,ab OR 'Overactive detrusor':ti,ab OR 'Urge incontinence':ti,ab OR 'Neurogenic bladder':ti,ab OR 'Detrusor hyperreflexia':ti,ab OR 'Detrusor overactivity':ti,ab OR 'Overactive bladder'/exp)

AND

(random* OR factorial OR crossover OR placebo OR blind OR blinded OR assign OR assigned OR allocate OR allocated OR 'crossover procedure'/exp OR 'double-blind procedure'/exp OR 'randomized controlled trial'/exp OR 'single-blind procedure'/exp) NOT ('animal'/exp NOT ('animal'/exp AND 'human'/exp))

**Cochrane CENTRAL**

(Darifenacin:ti,ab) AND ("Overactive Bladder":ti,ab OR "Overactive urinary bladder":ti,ab OR "Overactive detrusor":ti,ab OR "Urge incontinence":ti,ab OR "Neurogenic bladder":ti,ab OR "Detrusor hyperreflexia":ti,ab OR "Detrusor overactivity":ti,ab OR [mh "urinary bladder, overactive"])

**Supplementary Material 2**: GRADE assessment

| **Certainty assessment** | | | | | | | **N^o^ of patients** | | **Effect** | | **Certainty** |
| --- | --- | --- | --- | --- | --- | --- | --- | --- | --- | --- | --- |
| **N^o^ of studies** | **Study design** | **Risk of bias** | **Inconsistency** | **Indirectness** | **Imprecision** | **Other considerations** | **Darifenacin** | **Placebo** | **Relative (95% CI)** | **Absolute (95% CI)** |  |
| **Total Discontinuations** | | | | | | | | | | | |
| 7 | randomised trials | not serious | not serious | Not serious | serious^a^ | publication bias strongly suspected^b^ | 153/1663 (9.2%) | 88/870 (10.1%) | **RR 0.93** (0.72 to 1.20) | **7 fewer per 1,000** (from 28 fewer to 20 more) | ⨁⨁◯◯  Low^a,b^ |
| **Undefined Discontinuations** | | | | | | | | | | | |
| 7 | randomised trials | not serious | not serious | not serious | serious^a^ | publication bias strongly suspected^b^ | 55/1663 (3.3%) | 32/870 (3.7%) | **RR 0.85** (0.54 to 1.33) | **6 fewer per 1,000** (from 17 fewer to 12 more) | ⨁⨁◯◯ Low^a,b^ |
| **Dry mouth adverse events** | | | | | | | | | | | |
| 7 | randomised trials | not serious | not serious | not serious | serious^a^ | publication bias strongly suspected dose response gradient^b^ | 445/1572 (28.3%) | 54/855 (6.3%) | **RR 3.64** (2.54 to 5.22) | **167 more per 1,000** (from 97 more to 267 more) | ⨁⨁⨁◯ Moderate^a,b^ |
| **Constipation adverse events** | | | | | | | | | | | |
| 7 | randomised trials | not serious | not serious | not serious | not serious | publication bias strongly suspected dose response gradient^b^ | 314/1572 (20.0%) | 50/855 (5.8%) | **RR 2.85** (2.08 to 3.90) | **108 more per 1,000** (from 63 more to 170 more) | ⨁⨁⨁⨁ High^b^ |

**CI:** confidence interval; **RR:** risk ratio

**Explanations**

a. Downgraded once because of wide confidence intervals

b. Downgraded once because articles have been funded by pharmaceutical companies

**Supplementary Material 3**: Table of Excluded Studies

| **No.** | **Reference** | **Reason for exclusion** |
| --- | --- | --- |
| 1 | Abrams, P., Kelleher, C., Huels, J., Quebe-Fehling, E., Omar, M. A., & Steel, M. (2008). Clinical relevance of health-related quality of life outcomes with darifenacin. BJU International, 102(2). | Same population as an included study |
| 2 | Chapple, C., Steers, W., Norton, P., Millard, R., Kralidis, G., Glavind, K., & Abrams, P. (2005). A pooled analysis of three phase III studies to investigate the efficacy, tolerability and safety of darifenacin, a muscarinic M3 selective receptor antagonist, in the treatment of overactive bladder. BJU International, 95(7). | Pooled data of multiple included RCTs |
| 3 | Esin, E., Ergen, A., Cankurtaran, M., Yavuz, B. B., Halil, M., Ulger, Z., Yeşil, Y., Kuyumcu, M. E., Ozcan, M., Cankurtaran, E., & Ariogul, S. (2015). Influence of antimuscarinic therapy on cognitive functions and quality of life in geriatric patients treated for overactive bladder. Aging Ment Health, 19(3). | No placebo |
| 4 | Foote, J., Glavind, K., Kralidis, G., & Wyndaele, J. J. (2005). Treatment of overactive bladder in the older patient: Pooled analysis of three phase III studies of darifenacin, an M3 selective receptor antagonist. European Urology, 48(3). | Same population as included study |
| 5 | Haab, F., Corcos, J., Siami, P., Glavind, K., Dwyer, P., Steel, M., Kawakami, F., Lheritier, K., & Steers, W. D. (2006). Long-term treatment with darifenacin for overactive bladder: Results of a 2-year, open-label extension study. BJU International, 98(5). | No placebo |
| 6 | Hill, S., Elhilali, M., Millard, R. J., Dwyer, P. L., Lheritier, K., Kawakami, F. T., & Steel, M. (2007). Long-term darifenacin treatment for overactive bladder in patients aged 65 years and older: analysis of results from a 2-year, open-label extension study. Curr Med Res Opin, 23(11). | Same population as an included study |
| 7 | Kay, G., Crook, T., Rekeda, L., Lima, R., Ebinger, U., Arguinzoniz, M., & Steel, M. (2006). Differential effects of the antimuscarinic agents darifenacin and oxybutynin ER on memory in older subjects. Eur Urol, 50(2). | Subjects did not have OAB |
| 8 | Khullar, V., Foote, J., Larson-Peters, A., Seifu, Y., & Hruska, J. (2011). Darifenacin effectively relieved overactive bladder symptoms as early as 6-8 days of initiating treatment. International Urogynecology Journal and Pelvic Floor Dysfunction, 22. | Conference abstract |
| 9 | Khullar, V., Foote, J., Seifu, Y., & Egermark, M. (2011). Time-to-effect with darifenacin in overactive bladder: A pooled analysis. International Urogynecology Journal, 22(12). | Same population as an included study |
| 10 | Lipton, R. B., Kolodner, K., & Wesnes, K. (2005). Assessment of cognitive function of the elderly population: Effects of darifenacin. Journal of Urology, 173(2). | Subjects did not have OAB |
| 11 | Luis, V., Loyola, J. P., Melissa, C., Felipe, M., & San Martin, C. (2018). Efficacy of darifenacin use combined with pelvic floor physiotherapy in overactive bladder syndrome treatment in women. International Urogynecology Journal, 29. | No placebo and participants had concurrent illnesses |
| 12 | Newman, D., Larson-Peters, A., Seifu, Y., Aronstein, W., & Mongay, L. (2009). Impact of 'dry days' on quality of life in patients with 'wet' overactive bladder: Results of a pooled analysis of three phase III studies with darifenacin. Neurourology and Urodynamics, 28(7). | Conference abstract |
| 13 | Serra, D. B., Affrime, M. B., Bedigian, M. P., Greig, G., Milosavljev, S., Skerjanec, A., & Wang, Y. (2005). QT and QTc interval with standard and supratherapeutic doses of darifenacin, a muscarinic M3 selective receptor antagonist for the treatment of overactive bladder. Journal of Clinical Pharmacology, 45(9). | Subjects did not have OAB |

**Supplementary Material 4**: Risk of bias graph. Presented as percentages, this provides the review authors’ judgements about each risk of bias item across all included studies


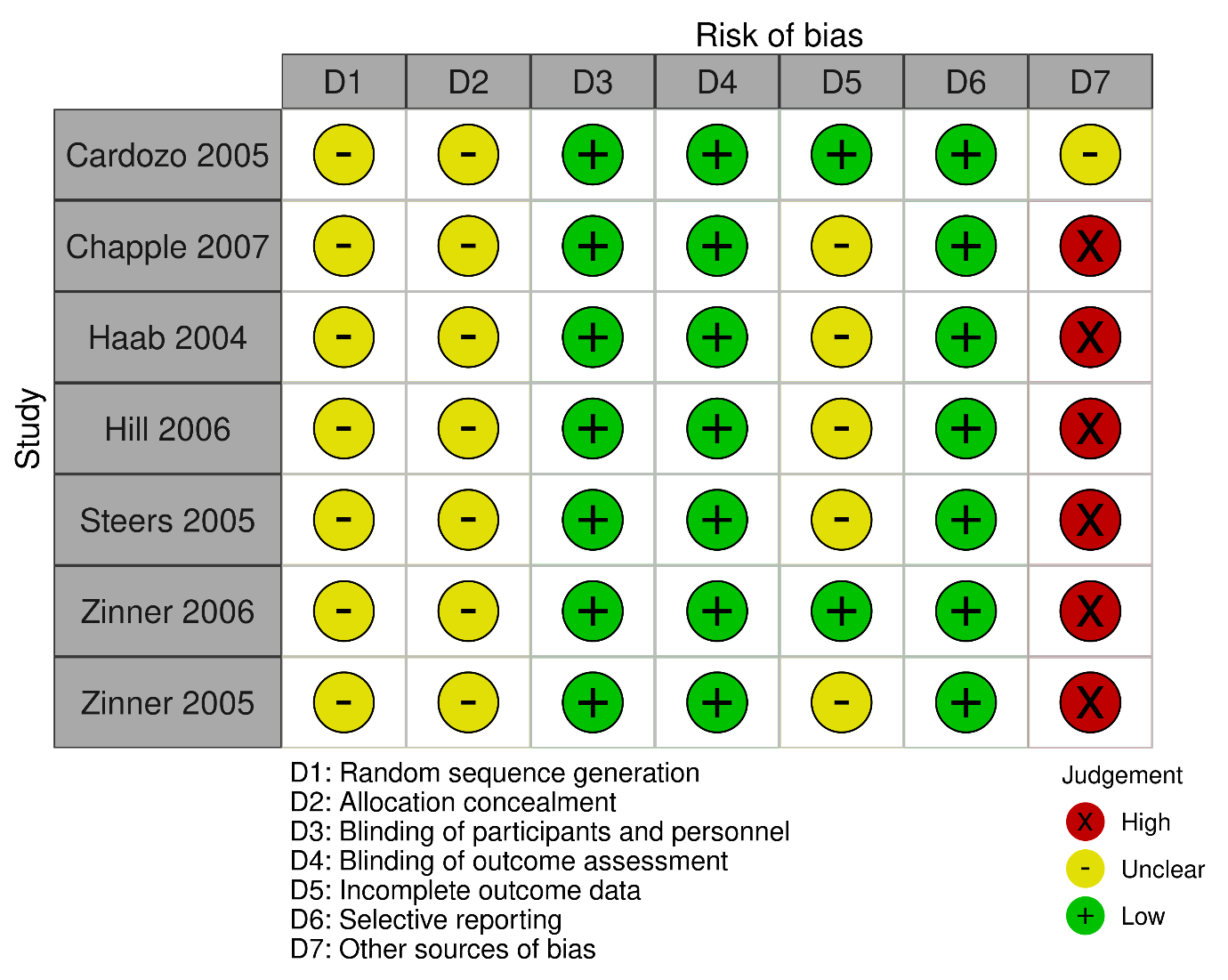


**Supplementary Material 5**: Dry mouth adverse event incidence of placebo and darifenacin, subgrouped by timepoint. CI, confidence interval.


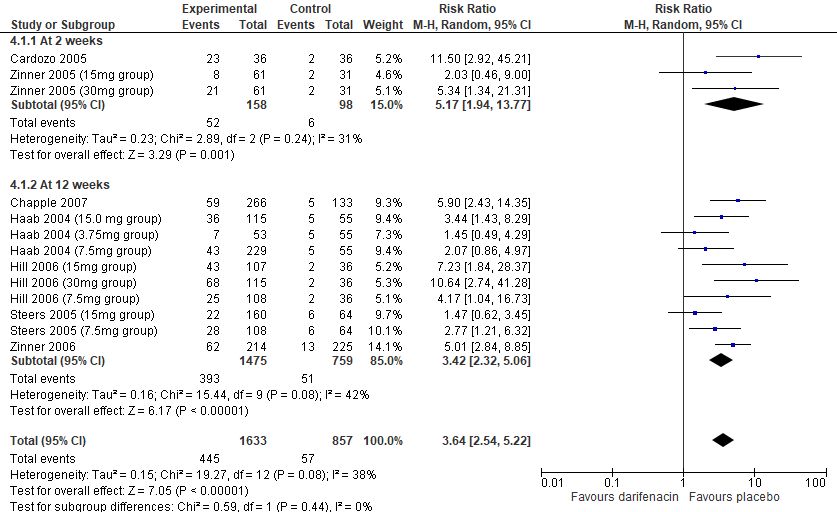


**Supplementary Material 6**: Constipation adverse event incidence of placebo and darifenacin, subgrouped by timepoint. CI, confidence interval.


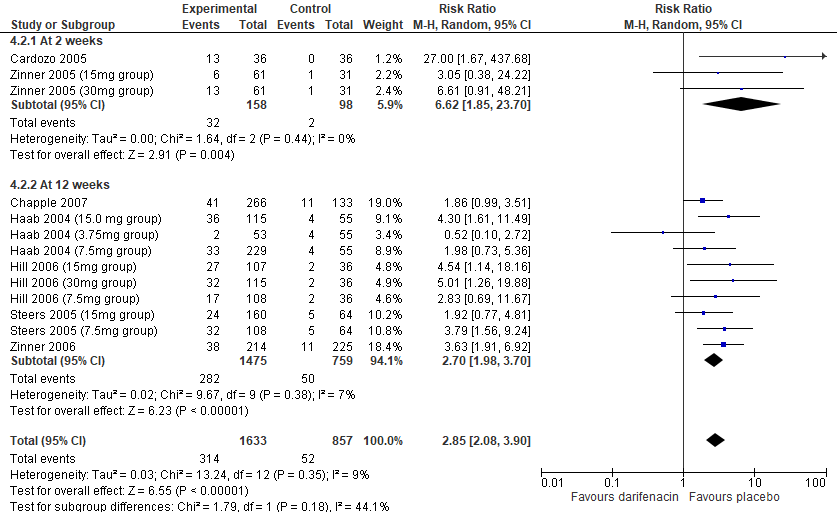


**Supplementary Material 7**: Total patient discontinuations from placebo and darifenacin, subgrouped by dose. CI, confidence interval.


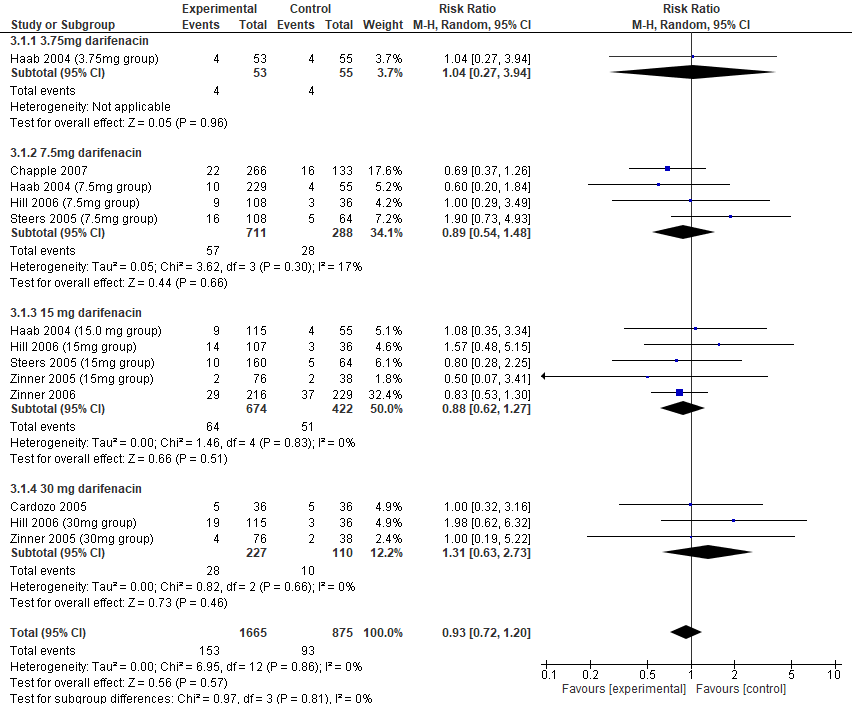


**Supplementary Material 8**: Undefined patient discontinuation from placebo and darifenacin, subgrouped by dose. CI, confidence interval.


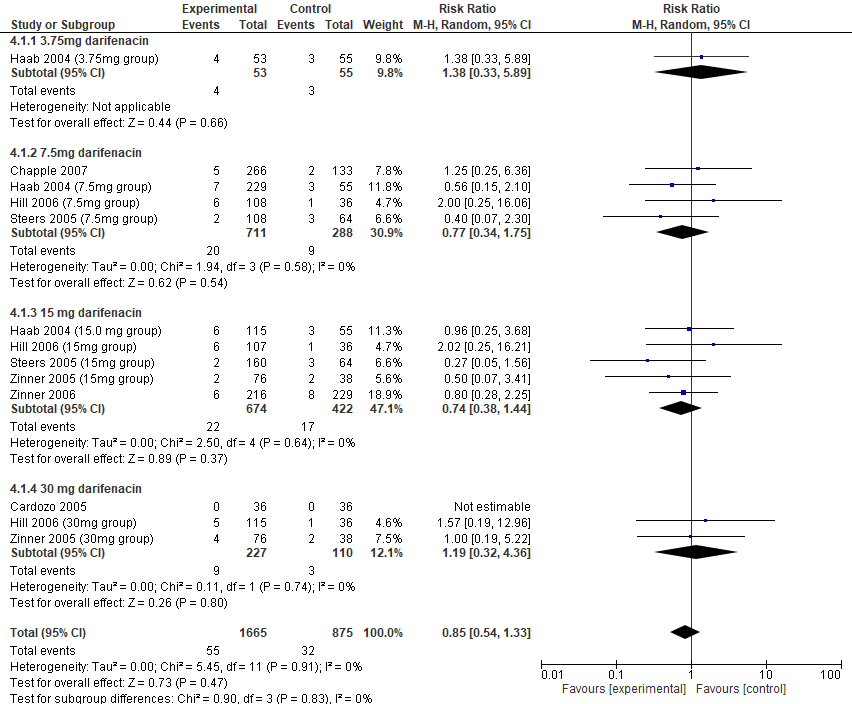


**Supplementary Material 9**: Undefined patient discontinuation from placebo and darifenacin, subgrouped by timepoint. CI, confidence interval.


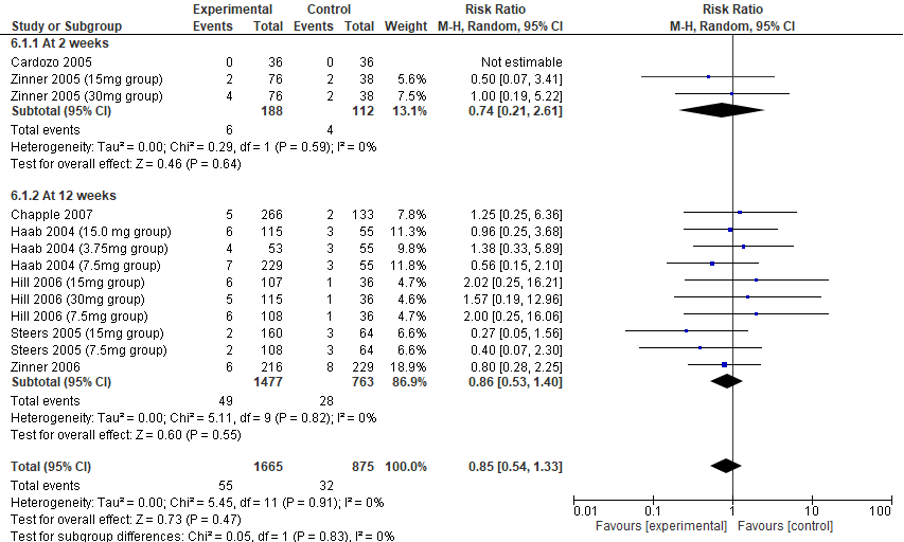


**Supplementary Material 10**: Adverse events for darifenacin and placebo comparator. All events are recorded, although separate columns of the three most commonly reported adverse events are provided for dry mouth and constipation. AE = adverse effects; NR = not reported.

| **Author**  **Year**  **Arm details** | **N (No. of participants)** | **Total AE (No. of participants)** | **Dry mouth AE (No. of participants)** | **Constipation AE (No. of participants)** | **Other adverse events (No. of participants)** |
| --- | --- | --- | --- | --- | --- |
| Cardozo  2005  A1: 30 mg Darifenacin  A2: Placebo | A1: 36 | A1: 27 (75.0%) | A1: 23 (63.9%) | A1: 13 (36.1%) | A1: Headache, 6 (16.7%). Dyspepsia, 4 (11.1%). Abnormal vision, 3 (8.3%). Dizziness, 2 (5.6%) |
|  | A2: 36 | A2: 3 (8.3%) | A2: 2 (5.6%) | A2: 0 | A2: No other adverse events |
| Chapple  2007  A1: 7.5 mg Darifenacin  A2: Placebo | A1: 266 | A1: 149 (56.0%) | A1: 59 (22.2%) | A1: 41 (15.4%) | A1: No other AE was reported by more than 5% of patients in either group. |
|  | A2: 133 | A2: 60 (45.1%) | A2: 5 (2.8%) | A2: 11 (8.3%) | A2: No other AE was reported by more than 5% of patients in either group. |
| Haab  2004  A1: 3.75 mg Darifenacin  A2: 7.5 mg Darifenacin  A3: 15 mg Darifenacin  A4: Placebo | A1: 53  A2: 229  A3: 115 | A1: 24 (45.3%)  A2: 120 (52.4%)  A3: 61 (53.0%) | A1: 7 (13.2%)  A2: 43 (18.8%)  A3: 36 (31.1%) | A1: 2 (3.8%)  A2: 33 (14.4%)  A3: 36 (31.3%) | A1: Headache, 1 (1.9%). Dyspepsia, 2 (3.8%), Gastritis 2 (3.8%)  A2: Headache, 2 (0.9%). Dyspepsia, 4 (1.7%), Gastritis 1 (0.4%)  A3: Headache, 4 (3.5%). Dyspepsia, 9 (7.8%), Gastritis 0 (0%) |
|  | A4: 164 | A4: 66 (40.2%) | A4: 14 (8.5%) | A4: 11 (6.7%) | A4: Headache, 4 (2.4%). Dyspepsia, 4 (2.4%), Gastritis 1 (0.6%) |
| Hill  2006  A1: 7.5mg darifenacin  A2: 15mg darifenacin  A3:30mg darifenacin  A4: Placebo | A1: 108  A2: 107  A3: 115 | A1: NR  A2: NR  A3: NR | A1: 25 (23.1%)  A2: 43 (40.2%)  A3: 68 (59.1%) | A1: 17 (15.7%)  A2: 27 (25.2%)  A3: 32 (27.8%) | A1: Headache, 7 (6.5%). Respiratory tract infection, 4 (3.7%). Urinary tract disorder, 0 (0%). Urinary tract infection, 3 (2.8%). Flu syndrome, 3 (2.8%). Back pain, 3 (2.8%). Abdominal pain, 1 (0.9%). Abnormal vision, 2 (1.9%).  A2: Headache, 7 (6.5%). Respiratory tract infection, 6 (5.6%). Urinary tract disorder, 6 (5.6%). Urinary tract infection, 3 (2.8%). Flu syndrome, 2 (1.9%). Back pain, 1 (0.9%). Abdominal pain, 3 (2.8%). Abnormal vision, 0 (0%).  A3: Headache, 7 (6.1%). Respiratory tract infection, 1 (0.9%). Urinary tract disorder, 1 (0.9%). Urinary tract infection, 5 (4.3%). Flu syndrome, 0 (0%). Back pain, 5 (4.3%). Abdominal pain, 4 (3.5%). Abnormal vision, 4 (3.5%). |
|  | A4: 109 | A4: NR | A4: 6 (5.5%) | A4: 5 (4.6%) | A4: Headache, 2 (1.8%). Respiratory tract infection, 6 (5.6%). Urinary tract disorder, 0 (0%). Urinary tract infection, 2 (1.8%). Flu syndrome, 5 (4.6%). Back pain, 3 (2.8%). Abdominal pain, 1 (0.9%). Abnormal vision, 0 (0%). |
| Steers  2005  A1:7.5 mg only darifenacin  A2: 7.5 mg uptitrated to 15 mg darifenacin  A3: placebo  A4: placebo with dummy uptitration | A1: 108  A2: 160 | A1/A2: 110 (41%) | A1: 28 (25.9%)  A2: 22 (13.75%) | A1: 32 (29.6%)  A2: 24 (15%) | A1/A2: Headache, 18 (7%). AEs of the nervous system (dizziness and somnolence), 10 (3.7%). AE of the cardiovascular system, 4 (1.5%). |
|  | A3: 41  A4: 86 | A3/A4: 26 (21%) | A3/A4: 11 (9%) | A3/A4: 10 (8%) | A3/A4: Headache, 7 (5.5%). AEs of the nervous system (dizziness and somnolence), 2 (1.6%). AE of the cardiovascular system, 1 (0.8%). |
| Zinner  2006  A1: 15 mg Darifenacin  A2: Placebo | A1: 214 out of 216 used for tolerability and safety profile. | A1: 136 (63.6%) | A1: 62 (29%) | A1: 38 (17.8%) | A1: Headache, 13 (6.1%). Urinary tract infection, 9.8%. Dyspepsia, 7.9%. Nausea, 3.7%. Diarrhea, 3.7%. |
|  | A2: 225 out of 229 used for tolerability and safety profile. | A2: 110 (48.9%) | A2: 13 (5.8%) | A2: 11 (4.9%) | A2: Headache, 5 (2.2%). Urinary tract infection, 8.4%. Dyspepsia, 2.2%. Nausea, 2.2%. Diarrhea, 4%. |
| Zinner  2005  A1: 15 mg Darifenacin  A2: 30 mg Darifenacin  A3: Placebo | A1: 61 out of 76 used for tolerability and safety profile.  A2: 61 out of 76 used for tolerability and safety profile. | A1: NR  A2: NR | A1: 8 (13.1%)  A2: 21 (34.4%) | A1: 6 (9.8%)  A2: 13 (21.3%) | A1: Blurred vision/dizziness.  A2: Blurred vision/dizziness, 0%. Deep vein  thrombophlebitis, cancer of the gall bladder, hypertension, exacerbation of hiatal hernia and cholecystitis (treatment unrelated), 1.6%. Fever and poorly differentiated adenocarcinoma (treatment unreleated), 1.6%. |
|  | A3: 61 out of 76 used for tolerability and safety profile. | A3: NR | A3: 3 (4.9%) | A3: 2 (3.3%) | A3: Blurred vision/dizziness, 0%. Menometrorrhagia and elective hysterectomy (treatment unrelated), 1.6%. |

**Supplementary Material 11**: Total and undefined OAB patient discontinuations of darifenacin treatment regime (w/d = withdrawal)

| **Author**  **Year**  **Arm details** | **Study**  **follow-up duration** | **N (No. of participants)** | **Total discontinuations in each arm (No. of participants)** | **Patient discontinuations accounted for by authors (No. of participants)** | **Reasons for patient discontinuation provided by authors** | **Patient discontinuations undefined for by authors (No. of participants)** |
| --- | --- | --- | --- | --- | --- | --- |
| Cardozo  2005  A1: 30 mg Darifenacin  A2: Placebo | 2 weeks | A1: 36 | A1: 5 (13.9%) | A1: 5 (13.9%) | A1: 4 w/d due to 1≥ symptoms of abnormal vision, dizziness, dry mouth, headache, paresthesia, constipation, nausea and dyspepsia and 1 w/d due to bladder carcinoma | A1: 0  (total darifenacin 0/36 [0%] undefined for) |
|  |  | A2: 36 | A2: 1 (2.8%) | A2: 1 (2.8%) | A2: 1 w/d due to protocol violation | A2: 0  (total placebo 0/36 [0%] undefined for) |
| Chapple  2007  A1: 7.5 mg Darifenacin  A2: Placebo | 12 weeks | A1: 266 | A1: 22 (8.3%) | A1: 17 (6.4%) | A1:12 w/d due to adverse events, 1 w/d due to administrative, 3 w/d due to lost in follow up and 1 w/d due to protocol violation | A1: 5 w/d due to withdrew consent  (total darifenacin 5/266 [1.9%] undefined for) |
|  |  | A2: 133 | A2: 16 (12.0%) | A2: 14 (10.5%) | A2: 9 discontinuations due to adverse events, 1 w/d due to administrative, 2 w/d due to protocol violation and 2 w/d due to unsatisfactory therapeutic effect | A2: 2 w/d due to withdrew consent  (total placebo 2/133 [1.5%] undefined for) |
| Haab  2004  A1: 3.75 mg Darifenacin  A2: 7.5 mg Darifenacin  A3: 15 mg Darifenacin  A4: Placebo | 12 weeks | A1: 53  A2: 229  A3: 115 | A1: 4 (7.5%)  A2: 10 (4.4%)  A3: 9 (7.8%) | A1: 0 (0%)  A2: 3 (1.3%)  A3: 3 (2.6%) | A2: 2 w/d due to constipation and 1 w/d due to rash.  A3: 1 w/d due to constipation, 1 w/d due to urticaria and tongue oedema and 1 w/d due to flatulence and insomnia. | A1: 4 w/d undefined for  A2: 7 w/d undefined for  A3: 6 w/d undefined for  (total darifenacin 17/397 [4.3%] undefined for) |
|  |  | A4: 164 | A4: 12 (7.3%) | A4: 2 (0.6%) | A4: 1 w/d due to urinary tract infection and 1 w/d due to constipation | A4: 10 w/d undefined for  (total placebo 10/164 [6.1%] undefined for) |
| Hill  2006  A1: 7.5mg darifenacin  A2: 15mg darifenacin  A3:30mg darifenacin  A4: Placebo | 12 weeks | A1: 108  A2: 107  A3: 115 | A1: 9 (8.3%)  A2: 14 (13.1%)  A3: 19 (16.5%) | A1: 3 (2.8%)  A2: 8 (7.5%)  A3: 14 (12.2%) | A1: 2 w/d due to adverse events and 1 w/d due to lack of response.  A2: 6 w/d due to adverse events and 2 w/d due to lack of response  A3: 13 w/d due to adverse events and 1 w/d due to lack of response. | A1: 6 w/d undefined for  A2: 6 w/d undefined for  A3: 5 w/d undefined for  (total darifenacin 17/330 [5.2%] undefined for) |
|  |  | A4: 109 | A4: 8 (7.3%) | A4: 5 (4.6%) | A4: 3 w/d due to adverse events and 2 w/d due to lack of response. | A4: 3 w/d undefined for  (total placebo 3/109 [2.8%] undefined for) |
| Steers  2005  A1:7.5 mg only darifenacin  A2:7.5 mg uptitrated to 15 mg darifenacin  A3: placebo  A4: placebo with dummy uptitration | 12 weeks | A1: 108  A2: 160 | A1: 16 (14.8%)  A2: 10 (6.3%) | A1: 14 (13.0%)  A2: 8 (5.0%) | A1: 12 w/d due to adverse events and 2 w/d due to protocol violation  A2: 6 w/d due to adverse events and 2 insufficient responses | A1: 2 w/d due to no longer wanting to continue.  A2: 1 w/d undefined for and 1 w/d due to no longer wanting to continue  (total darifenacin 4/268 [1.5%] undefined for) |
|  |  | A3: 41  A4: 86 | A3: 7 (17%)  A4: 3 (6%) | A3: 4 (9.8%)  A4: 1 (1.2%) | A3: 4 w/d due to adverse events  A4: 1 insufficient response | A3: 1 w/d undefined for, and 2 w/d due to no longer wanting to continue  A4: 2 w/d due to no longer wanting to continue  (total placebo 5/127 [3.9%] undefined for) |
| Zinner  2006  A1: 15 mg Darifenacin  A2: Placebo | 12 weeks | A1: 216 | A1: 29 (13.6%) | A1: 23 (10.7%) | A1: 17 w/d due to adverse events, 3 w/d due to protocol violations, 2 unsatisfactory therapeutic effects and 1 lost in follow-up | A1: 6 w/d due to withdrawn consent  (total placebo 6/214 [2.8%] undefined for) |
|  |  | A2: 229 | A2: 37 (16.4%) | A2: 29 (12.9%) | A1: 10 w/d due to adverse events, 9 w/d due to administrative, 4 w/d due to lost in follow up, 5 w/d due to protocol violation, and 1 abnormal test procedure | A2: 8 w/d due to withdrew consent  (total placebo 8/225 across all groups [3.6%] undefined for) |
| Zinner  2005  A1: 15 mg Darifenacin  A2: 30 mg Darifenacin  A3: Placebo | 2 weeks | A1: 76  A2: 76 | A1: 2 (2.6%)  A2: 4 (5.3%) | A1: 0  A2: 0 | Across all groups authors report that in most cases the reason for discontinuation was unrelated to treatment. | Lack of information for discontinuations in each group suggest all patient discontinuations are undefined for  (total across all groups 10/228 [4.4%] undefined for) |
|  |  | A3: 76 | A3: 4 (5.3%) | A3: 0 |  |  |
